# Fat-free noncontrast whole-heart cardiovascular magnetic resonance imaging with fast and power-optimized off-resonant water-excitation pulses

**DOI:** 10.1016/j.jocmr.2024.101096

**Published:** 2024-09-14

**Authors:** Adèle L.C. Mackowiak, Davide Piccini, Ruud B. van Heeswijk, Roger Hullin, Christoph Gräni, Jessica A.M. Bastiaansen

**Affiliations:** a Department of Diagnostic, Interventional and Pediatric Radiology (DIPR), Inselspital, Bern University Hospital, University of Bern, Bern, Switzerland; b Translation Imaging Center (TIC), Swiss Institute for Translational and Entrepreneurial Medicine, Bern, Switzerland; c Department of Radiology, Lausanne University Hospital (CHUV) and University of Lausanne (UNIL), Lausanne, Switzerland; d Advanced Clinical Imaging Technology (ACIT), Siemens Healthcare AG, Lausanne, Switzerland; e Department of Cardiology, Faculty of Biology and Medicine, Lausanne University Hospital, University of Lausanne, Lausanne, Switzerland; f Department of Cardiology, Inselspital, Bern University Hospital, University of Bern, Bern, Switzerland

**Keywords:** MRI, 3T, Fat signal suppression, RF excitation pulse, Off-resonant water excitation, SAR

## Abstract

**Background:**

Cardiovascular magnetic resonance imaging (CMR) faces challenges due to the interference of bright fat signals in visualizing structures, such as coronary arteries. Effective fat suppression is crucial, especially when using whole-heart CMR techniques. Conventional methods often fall short due to rapid fat signal recovery, leading to residual fat content hindering visualization. Water-selective off-resonant radiofrequency (RF) pulses have been proposed but come with tradeoffs between pulse duration, which increases scan time, and increased RF energy deposit, which limits their applicability due to specific absorption rate (SAR) constraints. The study introduces a lipid-insensitive binomial off-resonant (LIBOR) RF pulse, which addresses concerns about SAR and scan time, and aims to provide a comprehensive quantitative comparison with published off-resonant RF pulses for CMR at 3T.

**Methods:**

A short (1 ms) LIBOR pulse, with reduced RF power requirements, was developed and implemented in a free-breathing respiratory-self-navigated three-dimensional radial whole-heart CMR sequence at 3T. A binomial off-resonant rectangular (BORR) pulse with matched duration, as well as previously published lipid-insensitive binomial off-resonant excitation (LIBRE) pulses (1 and 2.2 ms), were implemented and optimized for fat suppression in numerical simulations and validated in volunteers (n = 3). Whole-heart CMR was performed in volunteers (n = 10) with all four pulses. The signal-to-noise ratio (SNR) of ventricular blood, skeletal muscle, myocardium, and subcutaneous fat and the coronary vessel detection rates and sharpness were compared.

**Results:**

Experimental results validated numerical findings and near-homogeneous fat suppression was achieved with all four pulses. Comparing the short RF pulses (1 ms), LIBOR reduced the RF power nearly two-fold compared with LIBRE, and three-fold compared with BORR, and LIBOR significantly decreased overall fat SNR from cardiac scans, compared to LIBRE and BORR. The reduction in RF pulse duration (from 2.2 to 1 ms) shortened the whole-heart acquisition from 8.5 to 7 min. No significant differences in coronary arteries detection and sharpness were found when comparing all four pulses.

**Conclusion:**

LIBOR pulses enabled whole-heart CMR under 7 min at 3T, with large volume fat signal suppression, while reducing RF power compared with LIBRE and BORR pulses. LIBOR is an excellent candidate to address SAR problems encountered in CMR sequences where fat suppression remains challenging and short RF pulses are required.

## Graphical abstract

**Figure:**
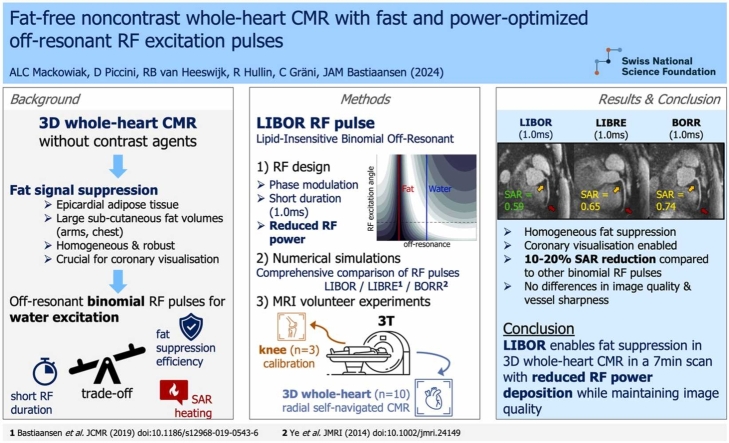

## 1 Introduction

In cardiac magnetic resonance imaging (CMR), bright fat signals hinder the visualization of small anatomical structures such as coronary arteries, which are embedded in adipose tissue [1]. When fat signal is not sufficiently suppressed, it can generate India ink artifacts contouring arteries [2] and can degrade overall image quality by the introduction of streaking artifacts [3], when subcutaneous chest fat is not sufficiently suppressed. Spoiled gradient recalled echo (GRE) sequences are the most commonly used acquisition method for anatomical CMR at 3T, but require good fat suppression notably in the absence of contrast agent. This essential fat signal suppression can prove to be especially challenging when using advanced whole-heart CMR techniques that employ non-Cartesian readout strategies [4], [5].

The rapid T1 recovery of the fat signal, following the application of conventional fat saturation, contributes to significant fat signal weighting in the k-space center of three-dimensional (3D) radial acquisitions, which results in cardiac images where fat remains largely present [3], albeit with lower signal intensity. Therefore, acquisition strategies are preferred that uniquely measure the water signal, either by using dedicated radiofrequency (RF) pulses for water-selective excitation [6] or by fast interruptions of balanced steady-state free precession (bSSFP) sequences [7], [8], [9].

Commonly used RF pulses for water excitation follow a binomial pattern, in which the spacing between the two subpulses is used to create a different excitation profile for both water and fat. This results in typical pulse durations on the order of 2 to 3 ms at 3T, which makes these RF pulses time-inefficient. By using a phase modulation on the second RF subpulse [10], [11], [12], [13], as for example done in a 1-90°-1 water-excitation pulse, the duration can be reduced by half compared with a conventional 1-180°-1 water-excitation pulse. However, the time savings come at the expense of a reduction in fat suppression bandwidth.

Excellent fat suppression capabilities have been reported for MR angiography when using a pair of binomial off-resonant rectangular (BORR) pulses with opposing phase. The BORR pulse [14], [15], developed by Ye et al., uses a 180° phase modulation on the second subpulse combined with a change in the RF excitation frequency. Although theoretically promising because of the wide fat suppression band it offers, a reduction of the BORR pulse duration below 2.6 ms has not been investigated.

The development of a lipid-insensitive binomial off-resonant excitation (LIBRE) RF pulse offered reduced pulse durations while outperforming the fat-suppressing capabilities of regular fat suppression methods and water-excitation approaches [16]. LIBRE pulses have durations that can be as short as 1 ms at 3T [17] and have been used for 3D radial whole-heart CMR at both 1.5T [18] and 3T [3], including free-running five-dimensional acquisitions [18], [19]. LIBRE pulses were successfully employed in coronary MR angiography in the clinical setting [19], [20], [21], enabling the detection of coronary aneurysms in children with Kawasaki disease at 3T [20]. Besides CMR, the pulses added value for musculoskeletal [17], [22], brain [23], and moving eye [24] imaging applications.

To reduce scan time, off-resonant pulse durations can be shortened by increasing the RF excitation frequency. However, this makes RF pulses less efficient in exciting on-resonant water. To mitigate this loss and achieve the desired water signal, the RF power needs to be increased, causing an increase in RF energy deposits (SAR). This poses practical limits on the use and flexibility of off-resonant excitation pulses where short repetition times (TR) are required [18]. No attempts have been made to optimize off-resonant water-excitation pulses in terms of RF power deposition, nor has a comprehensive comparison been conducted between the various off-resonant water-excitation pulses published to date.

The aim of the study was to implement a novel lipid-insensitive binomial off-resonant (LIBOR) RF pulse for water excitation which addresses concerns on RF energy deposit and total pulse duration. The LIBOR pulse was implemented for noncontrast free-breathing respiratory-self-navigated whole-heart CMR at 3T, within a 3D radial spoiled GRE sequence. With the additional implementation of LIBRE and BORR pulses of equivalent pulse duration (1 ms), a comprehensive quantitative comparison was provided between the different types of binomial off-resonant pulses for water excitation. Their fat suppression capabilities were quantified using numerical simulations and in healthy subjects.

## 2 Methods

### 2.1 RF pulse design of LIBOR

LIBOR was designed as a phase-modulated off-resonant water-excitation pulse with a total RF duration of 1 ms. LIBOR uses an excitation frequency of 780 Hz, which increases the water-excitation efficiency compared with a LIBRE pulse of the same duration [17]. The relatively low excitation frequency of LIBOR compared with LIBRE and BORR makes the LIBOR pulse by design more efficient in exciting water and thus requires a lower RF power. Then, similarly to phase modulation of a 1-90°-1 water-excitation pulse [12], the second LIBOR subpulse requires a phase offset to achieve fat signal suppression, which was determined using numerical simulations of the Bloch equations, which were additionally verified in magnetic resonance imaging (MRI) experiments. Simulations were performed to compute the transverse magnetization M_xy_ as a function of phase modulation and off-resonance after a single LIBOR RF excitation with an RF excitation angle of 10°. Off-resonance was varied between −800 Hz and 800 Hz in steps of 5 Hz and the phase modulation between 0° and 360° in steps of 5°. The phase modulation corresponding to the widest signal suppression band around fat, defined as 10% of the maximum observed transverse magnetization (M_xy_), was selected for subsequent measurements. All simulations were performed in MATLAB 2021 (The MathWorks, Natick, Massachusetts). The code is shared on our Github page.

### 2.2 Numerical simulations of LIBOR, LIBRE, and BORR pulses

Additional simulations were performed as described earlier [3], [16] to compute the transverse magnetization (M_xy_) of GRE sequences using a LIBOR, BORR, and LIBRE pulse of 1 ms duration. An additional LIBRE pulse of 2.2 ms duration was added for the comparison because this pulse has been investigated before for whole-heart radial CMR at 3T [3], in which it was evaluated against conventional fat suppression and water excitation. Conventionally, using an RF excitation angle of 10° typically refers to the rotation of the water magnetization with the same angle. However, this is not the case for off-resonant pulses, because the excitation frequency is not centered at the water frequency (0 Hz). Therefore, the RF excitation angle, which is set in the user interface of the scanner, needs to be increased to reflect the increase in RF power to achieve the actual desired rotation angle of the water magnetization or the nominal RF excitation angle.

The M_xy_ was determined as a function of RF excitation angle and off-resonance, providing a quantitative comparison of water-excitation efficiency, i.e., the required increase in power deposit relative to on-resonant pulses, fat signal suppression, and corresponding suppression bandwidths. RF excitation angles were varied between 2° and 30° in steps of 2°, the off-resonance was varied between −800 Hz and 800 Hz in steps of 5 Hz, and a TR of 5 ms, a T_1_ of 2000 ms, and a T_2_ of 50 ms were used. To ensure a steady state, 500 excitations were simulated with perfect RF and gradient spoiling by nulling M_xy_ after each RF excitation. The results of these simulations informed on RF power increases for subsequent MRI experiments. The RF waveforms corresponding to each off-resonant pulse were plotted for comparison.

### 2.3 MRI experiments

Volunteer experiments were performed on a 3T clinical MRI scanner (MAGNETOM Prisma^fit^, Siemens Healthcare, Erlangen, Germany) after obtaining written and informed consent from all participants. The study was approved by a local Ethics Committee (authorization CER-VD 2021-00708, Lausanne, Switzerland).

A LIBOR, BORR, and LIBRE RF pulse were implemented in a 3D radial spoiled GRE sequence following a spiral phyllotaxis trajectory [4], with the option to perform ECG-triggered and respiratory-self-navigated whole-heart acquisitions, as described in [3], [5]. The user interface of the scanner console was modified to select between the LIBOR, BORR, and LIBRE RF pulses by varying the duration (τ) of the subpulses, their RF excitation frequency (f_RF_), and the phase modulation of the second subpulse (ΔΦ) (Table 1). The research sequence can be requested on the exchange platform (TeamPlay) of the vendor.

**Table 1:** RF pulses properties with corresponding SAR values.

MR parameters	LIBOR (1.0 ms)	LIBRE (1.0 ms)	BORR (1.0 ms)	LIBRE (2.2 ms)
Excitation frequency f_RF_ (Hz)	780	**f**_**RF**_**= 1620**	**f**_**RF**_**= 1540**	**f**_**RF**_**= 520**
Subpulse duration τ (μs)	500	500	500	1100
Phases ${\text{Φ}}_{1}$, ${\text{Φ}}_{2}$	${\text{Φ}}_{2}=\;{\text{Φ}}_{1}+\;\Delta \Phi$ ΔΦ=320°	${\text{Φ}}_{2}=\;{\text{Φ}}_{1}+$ 2πτf_RF_	${\text{Φ}}_{2}=\;{\text{Φ}}_{1}+$ π	${\text{Φ}}_{2}=\;{\text{Φ}}_{1}+$ 2πτf_RF_

Knee experiments				
RF excitation angle* (deg) *Sequence parameter*	12	23	35	10
Nominal RF excitation angle* (deg) *Rotation of water*	6	6	6	6
SAR (mW/kg)	2.07	9.18	21.3	0.62

Cardiac experiments				
RF excitation angle* (deg) *Sequence parameter*	19	38	56	16
Nominal RF excitation angle* (deg) *Rotation of water*	10	10	10	10
SAR (W/kg)	0.59	0.65	0.74	0.58

Three types of off-resonant binomial RF pulses were tested in this study: LIBOR, BORR, and LIBRE. For LIBRE, a total RF pulse duration of 1 and 2.2 ms was used, which are RF pulses that have been used in prior studies [3], [16]. The RF excitation angle, the RF excitation frequency, the subpulses durations, and phase offset of each type of RF pulse are indicated. The tuning parameter and its value yielding optimal fat suppression for each pulse are highlighted in bold.

Data in this table are reported as numbers, with physical units indicated in the first column.

*RF* radiofrequency*, SAR* specific absorption rate*, LIBOR* lipid-insensitive binomial off-resonant*, LIBRE* lipid-insensitive binomial off-resonant excitation*, BORR* binomial off-resonant rectangular

* In this table, “RF excitation angle” refers to the sequence parameter that is chosen in the user interface of the sequence. Off-resonant pulses do not excite the water magnetization efficiently. Therefore, to ensure the same rotation angle of the water magnetization for each off-resonant pulse, i.e. the “nominal RF excitation angle,” the RF power needs to be adjusted. This is achieved by changing the RF excitation angle in the user interface.

Sequence parameters included an isotropic field-of-view of 200 mm^3^, a spatial resolution of 1.14 mm^3^, and a pixel bandwidth of 888 Hz/px. For the scans using LIBOR, BORR, and LIBRE RF pulses with a total duration of 1 ms, the echo time (TE) and TR were TE/TR = 1.99 ms/4.30 ms. For LIBRE scans with a total pulse duration of 2.2 ms, the TE/TR was 2.59 ms/5.53 ms. The RF excitation angles varied for each pulse because of their different RF power requirements. However, the nominal RF excitation angle, which refers to the rotation of the water magnetization, was equal for all pulses (Table 1). Image reconstruction for all experiments was performed at the scanner, and the SAR was recorded.

#### 2.3.1 RF pulse optimization in vivo

Knee experiments were performed to test the effect of varying the RF excitation frequency (LIBRE and BORR) or RF phase modulation (LIBOR) on the measured water and fat signals. The knees of n = 3 subjects were scanned using a 35-channel knee coil array. The 3D radial trajectory consisted of 513 spiral segments, which are successively rotated by the golden angle about the longitudinal axis. Each spiral segment was made of 24 readout lines that go from one periphery of the k-space to the opposite, while passing through the k-space center. This trajectory has been previously described mathematically and has been shown to provide a general reduction of eddy current artifacts and improvement in image quality [4].

The phase offset of LIBOR was varied from 270° to 340° in steps of 10°, the frequency f_RF_ of BORR and LIBRE (1 ms) was varied from 1500 to 1700 Hz in steps of 20 Hz, and the frequency f_RF_ of LIBRE (2.2 ms) was varied from 400 to 560 Hz in steps of 20 Hz.

The acquisition time was TA = 0:53 min per scan using LIBOR, BORR, and LIBRE (1 ms) pulses, and TA = 1:08 min per scan with LIBRE (2.2 ms) and was fixed across subjects.

#### 2.3.2 Noncontrast free-breathing respiratory-self-navigated whole-heart MRI at 3T

Free-breathing electrocardiogram (ECG)-triggered respiratory-self-navigated whole-heart MRI acquisitions were performed with 100% scan efficiency in n = 10 subjects (F = 6, M = 4, age [22; 34] years old) using an 18-channel chest coil array. The 3D radial spiral phyllotaxis pattern described in the previous section was used. This trajectory provides pseudo-random sampling non-uniformity as well as a repeated sample in the main orientation of the respiratory motion, two features that allow for respiratory-motion robust CMR [5]. A diastolic window for data collection was maintained at 100 ms per heartbeat. For the LIBOR, BORR, and LIBRE scans with a 1 ms total pulse duration, the acquisition was segmented into 424 spirals, each comprising 23 lines. For the LIBRE scans with the 2.2 ms total RF pulse duration, the trajectory was 18 lines and 547 spirals. This provided a matching 3D spiral pattern while keeping the same acquisition window for all scans, including those using a longer RF pulse. A vendor-provided T2 preparation module [25] of 40 ms was used to improve blood-myocardium contrast, which was played out with each heartbeat. The total amount of acquired k-space lines was the same for all scans (∼10k). Respiratory-motion-corrected cardiac images were reconstructed at the scanner, using a method for respiratory-self-navigation [26] based on blood pool identification and signal intensity variations in one-dimensional superior-inferior projections [5].

### 2.4 Image analysis

The digital imaging and communications in medicine images reconstructed at the scanner were analyzed by computing the signal-to-noise ratio (SNR) and contrast-to-noise ratio (CNR) in manually drawn regions of interest (ROIs) using the ImageJ software [27]. The brightness and contrast of all images were automatically matched in the software. ImageJ provided the area, mean, and standard deviation (SD) of the signal intensity measured in the ROIs.

The following definitions of SNR and CNR were used for all analyses presented in this study.

(1) $${\mathrm{SNR}}_{A}=\frac{\mathrm{mean}(S_{A})}{\mathrm{SD}(S_{\mathrm{bkg}})}$$

(2) CNR_A__−__B_ = SNR_A_ − SNR_B_

where $S_{A}$ is the signal intensity of tissue A, and $S_{\mathrm{bkg}}$ is the signal intensity in the background of the image.

#### 2.4.1 Knee MRI

ROIs corresponding to three tissue types (bone marrow, subcutaneous fat, and *vastus medialis* muscle) and a background region were drawn in five slices of each one of the four 3D datasets. The order of data analysis was randomized. SNR was reported as a mean and SD across all subjects. For each water-excitation pulse, the tuning parameter (RF excitation frequency or phase) that maximizes muscle-fat CNR (defined according to Eq. (2)) was chosen as the optimal RF parameter for that pulse and used in subsequent cardiac experiments.

#### 2.4.2 Whole-heart MRI

ROIs placed in the left-ventricular blood pool, the myocardium muscle, and the chest subcutaneous fat were drawn in each of the n = 10 volunteers. The ROIs were used to analyze the datasets obtained with the four water-excitation pulses, with adjustments in shape where necessary, and a randomized order of analysis. The SNR of the three tissue types, as well as the blood-fat CNR, was reported as mean and SD across all subjects.

All 3D whole-heart imaging volumes were analyzed for the detection and visualization of the right coronary artery (RCA), the left main (LM), and the left anterior descending (LAD) coronary arteries using the MR angiography analysis software SoapBubble [28]. Coronary reformats were obtained by tracking the vessels of interest. Vessel detection rates, as well as vessel sharpness along the first 4 cm when applicable, were reported.

#### 2.4.3 Statistical methods

All results are presented as mean ± SD of the mean. For SNR and CNR analyses, as well as for coronary sharpness analysis, statistically significant differences between the proposed LIBOR pulse and the three other water-excitation pulses were assessed using paired two-tailed Student’s t-tests using dedicated software (GraphPad Prism, San Diego, California), with a correction for multiple comparisons using the Bonferroni method. In all analyses, p < 0.05 was considered statistically significant.

## 3 Results

### 3.1 Numerical simulation experiments

**Fig. 1:**
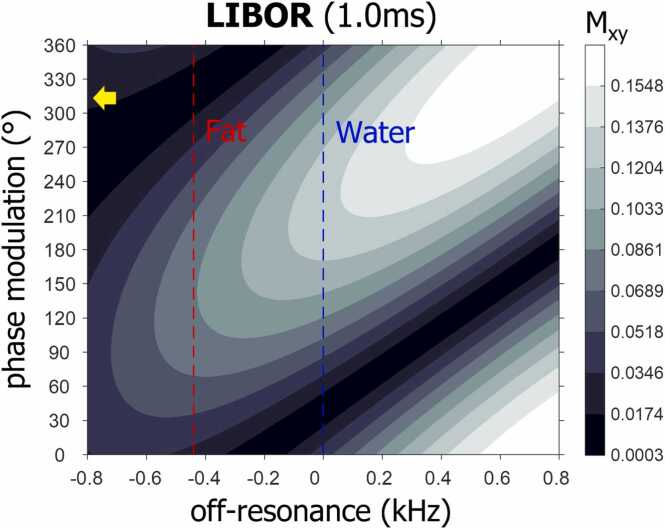
Frequency response profile of LIBOR. Frequency response profile of the LIBOR pulse as function of phase modulation applied to the second RF subpulse. Results were obtained following a numerical simulation of the transverse magnetization M_xy_ after a single LIBOR RF excitation of α = 10°. These results indicate that a phase modulation of about 315° (yellow arrow), equivalent to −45°, results in a fat suppression band around the expected fat resonance at −440 Hz. *LIBOR* lipid-insensitive binomial off-resonant, *RF* radiofrequency

**Fig. 2:**
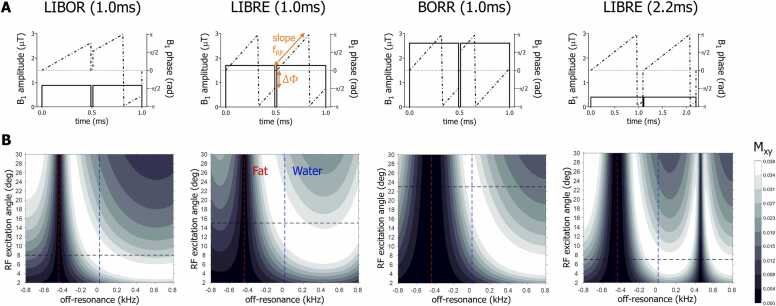
RF pulse shape and magnetization response. (A) RF pulse waveforms illustrating the B_1_ amplitude (solid line) and B_1_ phase (dashed line) for the LIBOR, LIBRE, BORR, and LIBRE (2.2 ms) pulse for maximized water excitation, which corresponds to the maximum observed M_xy_ for water (0 Hz) indicated in panel (B). The tuning parameters of the binomial RF pulses are labeled in orange on the LIBRE (1.0 ms): the water-excitation frequency f_RF_ corresponds to the slope of the B1 phase, while the phase offset ∆Φ corresponds to the difference in B1 phase between the end of the first subpulse and the start of the second subpulse (Table 1). (B) Bloch simulations of a GRE sequence with the four different off-resonant pulses illustrate the effect on the transverse magnetization M_xy_ as function of RF excitation angle and off-resonance (500 excitations, TR =5 ms, T_1_ = 2000 ms, T_2_ = 50 ms). These simulations depict the expected water (0 Hz, blue dashed line) and fat (−440 Hz, red dashed line) signal behavior, including the optimal RF excitation angle for water excitation (intersection of the black and blue dashed lines), which provides a relative measure of the required RF power in comparison with the other RF pulses. The intersection of the dashed red and black lines provides an indication of the fat suppression band. The optimal water M_xy_ for each pulse corresponds with the B_1_ amplitude in panel (A). The BORR pulse shows the widest fat suppression band but requires the highest B1 amplitude. *RF* radiofrequency, *LIBOR* lipid-insensitive binomial off-resonant, *LIBRE* lipid-insensitive binomial off-resonant excitation, *BORR* binomial off-resonant rectangular, GRE gradient recalled echo, *TR* repetition time

Simulation experiments show excellent fat suppression with a bandwidth of 300 Hz of the novel LIBOR pulse using a −45° phase modulation (Fig. 1), in comparison to a bandwidth of 130 Hz of a conventional 1-90°-1 pulse (data not shown). The simulated GRE signal attests to the different RF power requirements in terms of RF excitation angle increase (Fig. 2) and demonstrates similar B1 behavior of the different pulses. The LIBOR water-excitation efficiency is higher compared with the LIBRE and BORR pulses of equivalent duration and requires the lowest RF power increase compared with a LIBRE pulse of 2.2 ms. Although the BORR pulse achieved the widest suppression band (Fig. 2), it required the highest RF power increase.

### 3.2 3D radial MRI in the knee for RF pulse calibration and comparisons at 3T

The results of RF pulse calibration experiments can be found in the Supplemental Material of this paper. A phase modulation of ΔΦ = 320° (or −40°) for the LIBOR pulse achieved lowest fat SNR and highest muscle SNR (Supplemental Material Fig. S1), matching numerical findings. Shortening the BORR pulse to 1 ms was possible and the lowest fat SNR was obtained using an RF excitation frequency of ∼1540 Hz (Table 1). Using optimized RF parameters for each pulse, the average SAR was significantly reduced when using the LIBOR RF pulse (Table 1).

Qualitatively, homogeneous fat signal suppression was achieved in subcutaneous fat and bone marrow with all RF pulses, resulting in good visualization of the various muscles and cartilages (Supplemental Material Fig. S2). Some significant differences in SNR between LIBOR and the other binomial RF pulses were detected (notably in the *vastus medialis* muscle, see Supplemental Material Fig. S3), but did not affect the overall image quality which was similar across all tested pulses. The lowest bone marrow SNR was detected in LIBOR images, with only a statistical difference detected with the longer 2.2 ms LIBRE pulse.

The highest average muscle-fat CNR across subjects was 66.3 ± 13.8 for LIBOR, 54.0 ± 14.0 for LIBRE (1 ms), 34.4 ± 12.4 for BORR, and 61.1 ± 16.6 for LIBRE (2.2 ms), obtained with the respective optimal RF tuning parameters as follows: ΔΦ = 320° for LIBOR, f_RF_ = 1620 Hz for LIBRE (1 ms), f_RF_ = 1540 Hz for BORR and f_RF_ = 520 Hz for LIBRE (2.2 ms) (Table 1).

### 3.3 Noncontrast respiratory-self-navigated whole-heart MRI at 3 T

Free-breathing whole-heart MRI was performed with 100% scan efficiency in all volunteers in under 7 min. Acquisition times varied based on the subject’s heart rate (Table 2) but were on average reduced by 20% using the 1 ms RF pulses. Recorded SAR values were lowest using the LIBOR pulse, when comparing the other RF pulses of short duration (Table 2).

**Table 2:** Detection and sharpness of cardiac vessels in volunteer experiments.

	LIBOR (1.0 ms)	LIBRE (1.0 ms)	BORR (1.0 ms)	LIBRE (2.2 ms)
Average TA (min:s)	6:45 ± 0:48	6:47 ± 0:52	6:57 ± 0:47	8:34 ± 1:00

*RCA*				
Detection rate	9/10	9/10	8/10	9/10
% vessel sharpness (first 4 cm)	36.5 ± 5.6	36.1 ± 7.6	33.9 ± 8.1	36.1 ± 6.8

*LM+LAD*				
Detection rate	9/10	9/10	8/10	9/10
% vessel sharpness (first 4 cm)	29.9 ± 6.3	28.9 ± 5.9	26.6 ± 4.9	27.2 ± 6.4

*LCX*				
Detection rate	7/10	7/10	6/10	7/10

Whole-heart free-breathing cardiac MRI performed using off-resonant LIBOR, BORR, and LIBRE pulses were analyzed for the detection of the right coronary artery (RCA), the left main (LM) and the left anterior descending (LAD) coronary artery, and the left circumflex (LCX) coronary artery. The percentage of vessel sharpness along the first 4 cm of the vessel (when applicable) was measured and reported as an average and standard deviation across volunteers. Specific absorption rate values and average scan time (TA) obtained with each pulse are indicated in the first two rows. While the 1-ms-long RF pulses provided a significant scan time acceleration compared with the 2.2-ms LIRBE pulse sequence, no significant differences in percentage of vessel sharpness were reported

Average TA and % vessel sharpness values are reported as means ± standard deviations. Detection rates are reported as number of cases (x/10 volunteers).

*MRI* magnetic resonance imaging*, LIBOR* lipid-insensitive binomial off-resonant*, LIBRE* lipid-insensitive binomial off-resonant excitation*, BORR* binomial off-resonant rectangular

**Fig. 3:**
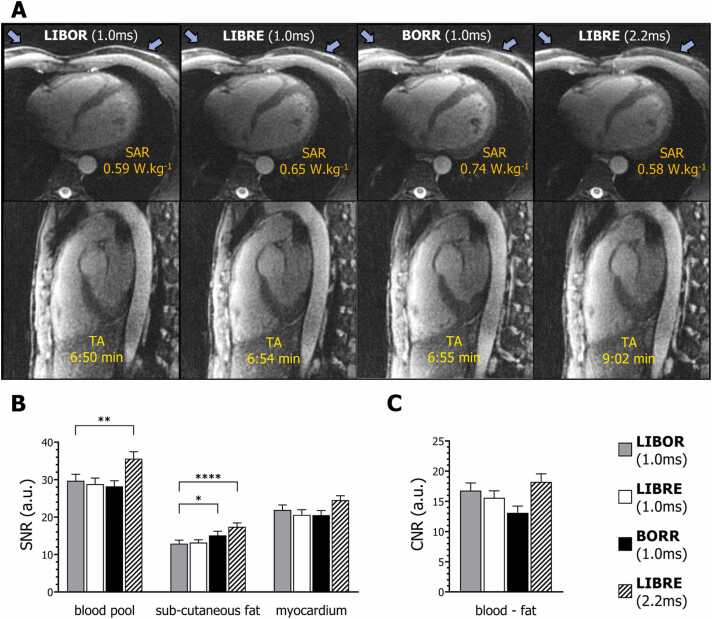
Free-breathing respiratory-self-navigated whole-heart MRI using different off-resonant water-excitation pulses. Transversal and sagittal views of 3D cardiac MR images in a volunteer, obtained with LIBOR, BORR, LIBRE (1 ms), and LIBRE (2.2 ms) are shown in (A). Fat signal suppression is nearly identical comparing the different RF pulses, but LIBOR maintains a short TR and simultaneously reduces SAR values. Slight variations in signal suppression of subcutaneous fat (A, blue arrows) could be observed, and statistically significant differences were found in SNR (B) and CNR (C) analysis. The acquisition times and SAR values are specified for each utilized RF pulse. SAR values are predominantly influenced by the power-demanding T2 preparation module, which was used to generate contrast between ventricular blood and myocardium in the T1-weighted GRE sequence [25]. *MRI* magnetic resonance imaging, 3D three-dimensional, *MR* magnetic resonance, *RF* radiofrequency, *LIBOR* lipid-insensitive binomial off-resonant, *LIBRE* lipid-insensitive binomial off-resonant excitation, *BORR* binomial off-resonant rectangular, *SAR* specific absorption rate, *SNR* signal-to-noise ratio, *CNR* contrast-to-noise ratio, *GRE* gradient recalled echo

Visually, all pulses performed well for fat suppression (Fig. 3A), with LIBOR demonstrating more homogenous fat suppression of subcutaneous chest fat (Fig. 3A, blue arrows). Overall, the SNR measurements on LIBOR whole-heart images were comparable to that of the other 1 ms RF pulses, but consistent significant differences were found when comparing it to the 2.2 ms LIBRE pulse (Fig. 3B) in both the blood pool (p < 0.01) and the subcutaneous fat (p < 0.0001). LIBOR provided the lowest subcutaneous fat SNR (Fig. 3B), with a value of 12.8 ± 0.9 across volunteers, comparable with that of the short 1 ms LIBRE pulse 13.2 ± 0.7. No statistically significant differences were observed in myocardium SNR, nor in blood-fat CNR, between all water-excitation RF pulses (Fig. 3C).

**Fig. 4:**
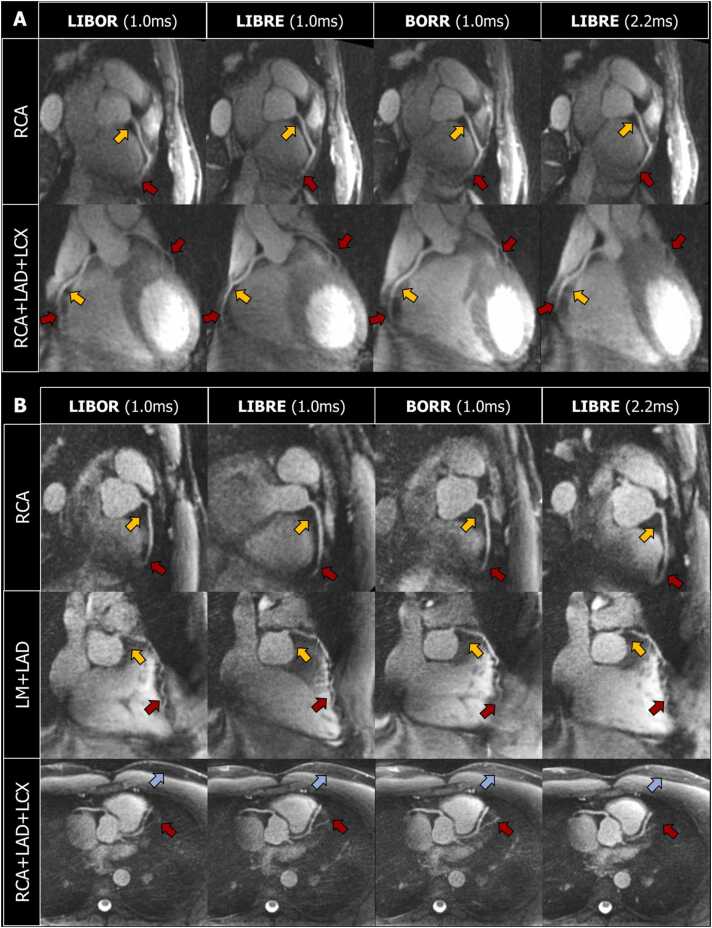
Coronary reformats in two volunteers. CMRA reformatted images from two volunteers (panels A and B) obtained with SoapBubble [28]. Transversal and coronal reformats were produced to visualize the right coronary artery (RCA), the left main (LM), and left anterior descending (LAD) arteries, as well as the left circumflex (LCX) coronary artery, when visible. Red arrows indicate (expected) coronary artery edges, yellow arrows indicate epicardial fat regions, and blue arrows indicate subcutaneous chest fat. *LIBOR* lipid-insensitive binomial off-resonant, *LIBRE* lipid-insensitive binomial off-resonant excitation, *BORR* binomial off-resonant rectangular

The coronary arteries could be visualized using all four water-excitation pulses (Fig. 4), with matching detection rates in all cases, with the RCA being detected in all subjects, the LM and LAD being detected in the same four of five subjects and the LCX being detected in the same three of five subjects (Table 2). In the subject where only the RCA was detected, unsuccessful respiratory-self-navigation caused residual respiratory motion artifacts, leading to a decreased image quality and difficulties to produce coronary reformats. A manual adjustment of the window for self-navigation was required in some subjects to accurately correct for respiratory motion (Supplemental Material Fig. S4). This, however, affected all the scans of this subject equally and, therefore, did not affect the comparison between the four water-excitation pulses.

Only minor differences could be observed in the coronary reformats, and the overall visualization of the coronaries was similar with all tested pulses, leading to the same expected edge and location of the vessel (Fig. 4). In particular, in the mid to distal portion of the LAD where achieving fat suppression homogeneity can be challenging, LIBOR was shown to exhibit similar performance as the other pulses.

While LIBOR provided higher vessel sharpness on the first 4 cm of the RCA than BORR, LIBRE (1 ms), and LIBRE (2.2 ms), statistical comparisons between LIBOR and the other three water-excitation pulses were all found to be non-significant (Table 2).

## 4 Discussion

In this study, a novel off-resonant RF pulse for water excitation, LIBOR, was designed and optimized for noncontrast whole-heart CMR at 3T. Effective and fast lipid signal suppression over large volumes was obtained with reduced RF power and thus reduced SAR deposits.

In comparison with published off-resonant RF pulse designs such as BORR [14] and LIBRE [16], the proposed LIBOR technique utilizes a phase modulation on the second subpulse to reduce signal coming from fatty tissues. By design, the different RF pulses appear similar, but the LIBOR RF excitation frequency is halved compared with LIBRE and BORR, which reduces the required RF power to excite water to the same extent as the other RF pulses. The optimal LIBOR phase modulation was found through numerical simulations and was experimentally verified.

A characteristic feature of off-resonance water-excitation pulses is their flexible pulse duration [16], which can be reduced at the expense of RF power. In the present study, it was observed that when the durations of the LIBRE, BORR, and LIBOR pulses were reduced to 1 ms, the RF power was highest for the BORR pulse and lowest for the LIBOR pulse. Interestingly, the BORR pulse was not the most off-resonant in terms of excitation frequency. This highlights that the behavior of RF pulses is not always straightforward, and even if RF pulse designs appear similar, their performances can differ. The anticipated decrease in RF power and SAR deposits with the LIBOR pulse was validated by in vivo experiments. It is important to note that the SAR values recorded during cardiac MRI experiments were predominantly influenced by the power-demanding T2 preparation module used to generate blood-to-myocardium contrast in the T1-weighted GRE sequence [25].

Although the BORR pulse had the highest RF power requirement, it offered the widest fat suppression band. So far, it was not investigated whether a shortening of the BORR pulse duration was possible. Prior work utilized BORR pulses with durations of 2.6 ms and an RF excitation frequency of 300 Hz [14], [15]. With these published settings, the RF power increases are minimal. This observation suggests that the use of BORR pulses could benefit acquisitions where scan time is less important and where TR can thus be increased. In this case, the BORR pulse may offer great performance in terms of fat signal suppression and water excitation. For sequences requiring a short TR, BORR would be a less ideal candidate. This is strengthened by observations of signal smearing in vivo when a 1 ms BORR pulse was used, likely caused by the high RF power requirement in combination with off-resonance effects. Similar signal bleeds, albeit to a lesser extent, were observed for the 1 ms LIBRE pulse.

Experiments on the human knee corroborated findings from numerical simulations and were utilized to select the optimal parameters for each type of RF pulse, ensuring a fair comparison among them. However, small deviations in RF pulse parameters have minimal impact on the final results. In all in vivo experiments, the parameters for the LIBRE pulses were consistent with those identified in previous studies conducted years ago on the same MRI scanner (LIBRE 1 ms [17], LIBRE 2.2 ms [3], [16]). This underscores that RF pulses do not necessitate dedicated fine-tuning when implemented and operated on different MRI scanners. Such fine-tuning was not necessary when a version of the whole-heart CMR sequence that uses LIBRE pulses [3] was installed and utilized at different clinical sites [19], [20], [21].

For whole-heart CMR, the use of short water-excitation pulses of 1 ms reduced the average scan time significantly, from 8:29 to 6:50 min, while neither image quality nor coronary artery visualization was significantly affected. Detection rates and observed vessel sharpness were similar for all tested RF excitation pulses, as expected. Although the theoretical fat suppression bands varied for different RF pulses tested in the current study, fat signal suppression was visually similar in all images. The study did not utilize advanced motion compensation or image reconstruction techniques based on compressed sensing, and images were directly reconstructed at the scanner with an inline respiratory-motion correction, provided by the vendor [5]. Therefore, the cardiac and coronary image quality could potentially be improved when using advanced image reconstruction methods [29], [30]. To facilitate research collaborations in this direction, the anonymized raw data, as well as the code to read it, are made available to the research community in a public repository.

The benefits of using off-resonant water-excitation pulses in 3D radial free-breathing respiratory-self-navigated whole-heart MRI have been reported in our prior work [3], notably within the frame of a comparison between 2.2 ms LIBRE pulses and conventional fat suppression methods. In this prior work, LIBRE outperformed conventional methods and significantly improved visualization of coronary arteries, due to improved fat signal suppression, which in turn had a beneficial effect on respiratory-self-navigation and helped reduce streaking artifacts. Therefore, the current study focused on a comprehensive quantitative comparison between different off-resonant RF excitation pulses.

The LIBOR pulse significantly reduces RF power deposits and offers a short TR, making it potentially ideal for acquisitions where a short TR is essential and where the acquisition is SAR intensive, such as with bSSFP sequences. Offering a short TR becomes even more important when lowering the magnetic field strengths, for example to 1.5T and below. At lower magnetic field strengths, water and fat have smaller frequency differences compared with 3T, which typically translates into an increased duration of water-excitation pulses. For example, to suppress fat signals with acceptable TR during whole-heart bSSFP acquisitions at 1.5T using LIBRE, a nominal RF excitation angle of approximately 120° was chosen [18]. However, this choice was influenced by the imposed SAR limit, which limited our ability to achieve the optimal water excitation and, consequently, the water SNR. Utilizing LIBOR RF pulses for whole-heart CMR at 1.5T, or at even lower magnetic field strengths such as 0.55T, could be a promising approach to address SAR issues and challenges in fat signal suppression. Notably, the SAR reduction provided by LIBOR would be of particular interest in the context of clinical CMR, where higher spatial resolutions are typically used (i.e., below 1 mm^3^) for coronary visualization, lengthening acquisition times. A comparative study between off-resonant RF pulses and various methods for fat signal suppression in whole-heart acquisitions, such as fast-interrupted steady-state sequences [7], [8], [9], the application of fat-suppressing T2 preparation modules [31], [32], or whole-heart water-fat separation [33] techniques, is a topic of ongoing research [34].

Homogenous fat signal suppression is challenging in large volumes, and even more so for non-Cartesian whole-heart MRI acquisitions. A comprehensive comparison was made between different off-resonant RF water-excitation pulses for their use in CMR at 3T. This included the development of a novel LIBOR pulse and the shortening of the BORR pulse. The successful implementation and comparison of LIBOR, BORR, and LIBRE pulses in a 3D radial sequence for noncontrast respiratory-self-navigated whole-heart MRI reveal that LIBOR is the most promising off-resonant water-excitation pulse for fat signal suppression in large volumes with reduced SAR.

## 5 Limitations

The volunteer cohort consisted of n = 10 healthy subjects, further validation should be performed in a patient cohort. Although the image reconstruction was performed directly at the scanner, the implementation for motion compensation occasionally failed (Supplemental Material F. S4). Therefore, image quality could be further improved using an off-line reconstruction. Finally, because this study focused on the SAR deposition of water-excitation pulses, the newly designed LIBOR pulse was compared only to other binomial water-excitation RF pulses.

## 6 Conclusion

A novel LIBOR RF pulse was developed for fast water excitation and was implemented in a 3D radial phyllotaxis GRE sequence enabling a noncontrast free-breathing respiratory-self-navigated whole-heart MRI acquisition in under 7 min. LIBOR demonstrated homogeneous fat suppression while reducing RF power and SAR compared with off-resonant pulses, such as LIBRE and BORR. These findings are especially interesting in addressing SAR problems encountered in MRI sequences where fat suppression remains challenging.

## Funding

This study was supported by funding received by Dr. Bastiaansen from the 10.13039/501100001711Swiss National Science Foundation (grants #PCEFP2_194296, #PZ00P3_67871), the University of Lausanne Bourse Pro-Femmes, the Emma Muschamp Foundation, and the 10.13039/501100004362Swiss Heart Foundation (grant #FF18054). Dr. Gräni received funding from the Swiss National Science Foundation, InnoSuisse, Center for Artificial Intelligence in Medicine University Bern, GAMBIT Foundation, 10.13039/501100004784Novartis Foundation for Medical-Biological Research, Swiss Heart Foundation, outside of the submitted work.

## Author contributions

**Jessica A. M. Bastiaansen:** Writing – review and editing, Writing – original draft, Visualization, Validation, Supervision, Software, Resources, Project administration, Methodology, Investigation, Funding acquisition, Formal analysis, Data curation, Conceptualization. **Adèle Mackowiak:** Writing – review and editing, Writing – original draft, Visualization, Validation, Software, Project administration, Methodology, Investigation, Formal analysis, Data curation. **Davide Piccini:** Writing – review and editing, Software, Resources, Methodology, Investigation, Conceptualization. **Ruud B. van Heeswijk:** Resources, Project administration, Investigation. **Roger Hullin:** Resources, Project administration, Investigation. **Christoph Gräni:** Resources, Project administration, Investigation.

## Declaration of competing interests

Dr. Piccini is an employee of Siemens Healthcare AG. Dr. Gräni serves as Editor-in-Chief of The International Journal of Cardiovascular Imaging, Springer. The authors declare that they have no known competing financial interests or personal relationships that could have appeared to influence the work reported in this paper.

## Data Availability

An online repository containing the anonymized human MRI raw data, as well as RF pulse shapes used in this study, is publicly available at: https://zenodo.org/records/8338079 (Part 1: KNEE V1-V3, HEART V1-V5). https://zenodo.org/records/10715769 (Part 2: HEART V6-V10). Matlab code to 1) simulate the different RF pulses within a gradient recalled echo sequence and 2) to read and display the anonymized raw data are available from: https://github.com/QIS-MRI/LIBOR_LIBRE_BORR_SimulationCode. The compiled research sequence can be requested through the Teamplay platform of Siemens Healthineers.
