# Supplementary material for: Fat-free noncontrast whole-heart cardiovascular magnetic resonance imaging with fast and power-optimized off-resonant water-excitation pulses
Source: J Cardiovasc Magn Reson. 2024 Sep 14;26(2):101096. doi: 10.1016/j.jocmr.2024.101096 (PMC11616052; doi:10.1016/j.jocmr.2024.101096)
Supplement: Supplementary file 1 — Supplementary material [file mmc1.docx]

# SUPPLEMENTAL MATERIAL


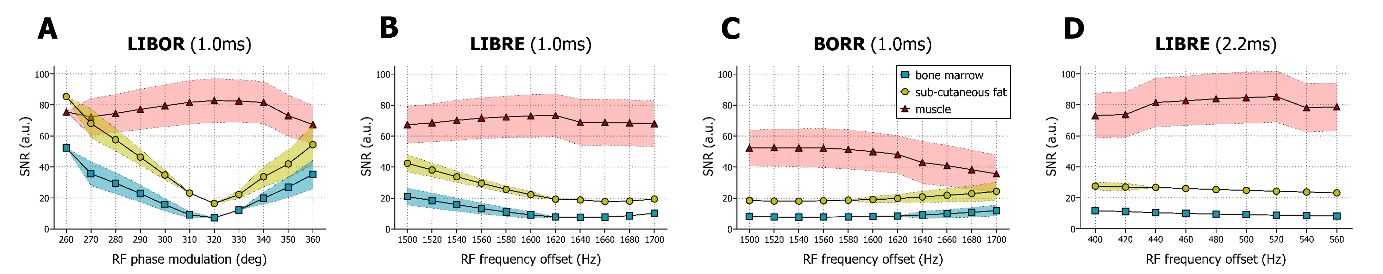


## Figure S1 – SNR of knee tissues in response to variation in RF frequency or phase excitation for the 4 water excitation pulses

The SNR in three ROIs located respectively in bone marrow, sub-cutaneous fat, and muscle (*vastus medialis*) was measured in 5 slices of a 3D acquisition using different off-resonant RF excitation pulses. The average and standard deviation across slices and volunteers (n=3) are plotted for each pulse as a function of its tuning parameter: RF phase modulation for LIBOR (A) and RF frequency offset for BORR (C) and LIBRE (B, D). An RF phase modulation of ~320° of the second LIBOR subpulse results in the highest fat signal suppression in subcutaneous fat and bone marrow, which is in agreement with a phase modulation of 315° obtained in numerical simulations (Fig.1). The drop in SNR of muscle tissue using the BORR pulse is most likely caused by an increase in background noise, which can be observed in the corresponding the knee images (Fig.S2).

## Figure S2 – Image comparison of binomial off-resonant RF pulses in knees

The fat-suppressed 3D MR images of the knee of research volunteer obtained with LIBOR, LIBRE (1ms), BORR, and LIBRE (2.2ms) are shown in transversal, coronal, and sagittal orientations. Although the suppression bandwidths were different for each pulse, the suppression of fat signal in the human knee was comparable. A close-up image including the lateral meniscus is shown. BORR images exhibit increased signal bleed in air-interfaced regions (green arrows).


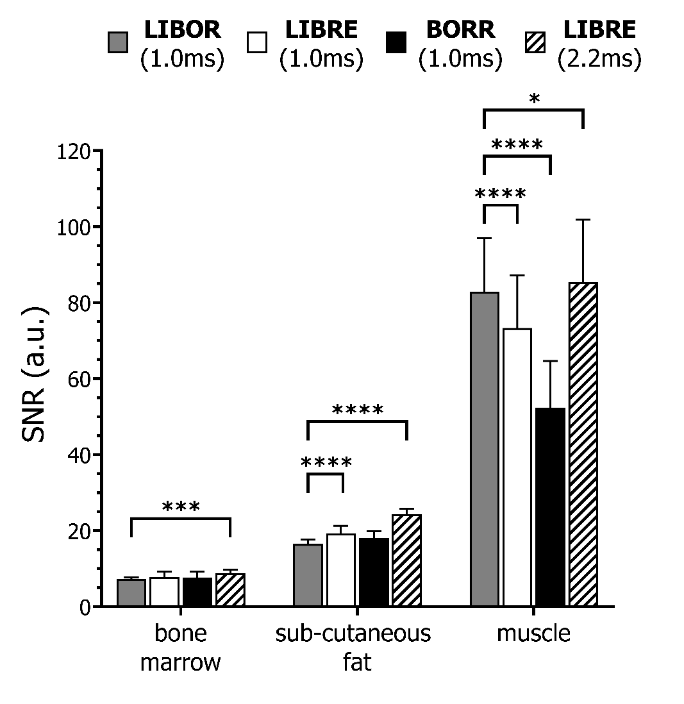


## Figure S3 – SNR comparison of binomial off-resonant RF pulses in knees

The average SNR and standard deviation across subjects in three tissue types (bone marrow, sub-cutaneous fat, and muscle) was computed for each of the four pulses. Significant differences between LIBOR and the other water excitation pulses are indicated with asterisks (*).

In bone marrow, a significant signal decrease was detected between LIBOR (7.2±0.4) and the 2.2ms-long LIBRE pulse (8.8±0.9, p<.0001). In sub-cutaneous fat, significant SNR decreases were detected between LIBOR (16.5±1.1) and LIBRE (1ms) (19.2±2.0, p<.0001) and between LIBOR and LIBRE (2.2ms) (24.3±1.5, p<.0001). The only significant difference in SNR between LIBOR and BORR was detected in the muscle (82.8±14.1 vs 52.4±12.2, p<.00001).

The drop in SNR of muscle tissue using the BORR pulse is likely caused by an increase of background noise, which is evident in the knee images in Fig.S2 (green arrows). The increase in muscle SNR with the LIBRE (2.2ms) pulse may be related to the longer TR used in this experiment.

## Figure S4 – Typical artifacts observed when the search position is incorrectly set for self-navigation for respiratory motion correction


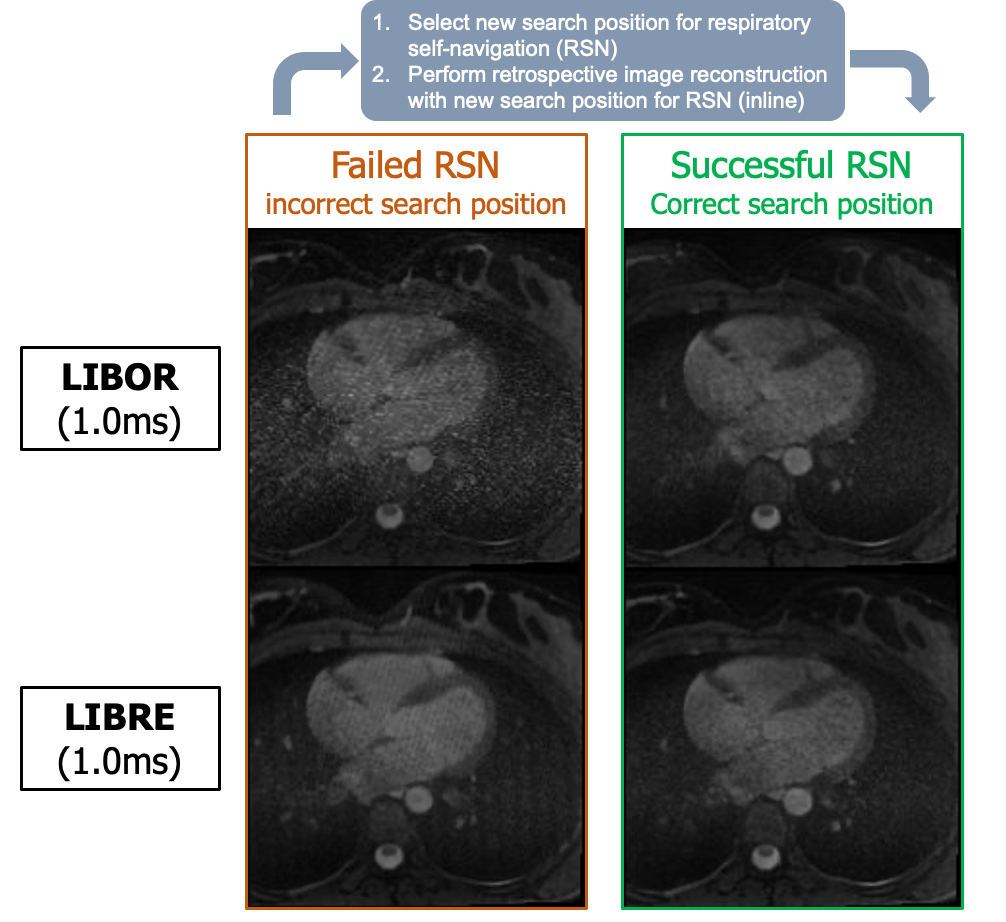


The importance of adjusting the search position of the self-navigation window is shown in one volunteer for two types of radial self-navigated (RSN) scans, with LIBOR (1.0ms) and LIBRE (1.0ms). On the left, the search position of the self-navigation window was not aligned with the location of the blood pool, leading to artefacts in the images. Following a straightforward retrospective image reconstruction performed on the scanner after adjusting the search position, the artifacts are resolved and motion correction is performed as intended (right panel).

More details on the implementation of RSN can be found in the original publication by Piccini et. al. (<https://onlinelibrary.wiley.com/doi/full/10.1002/mrm.23247>).
